# The flavonoid, naringenin, decreases adipose tissue mass and attenuates ovariectomy-associated metabolic disturbances in mice

**DOI:** 10.1186/1743-7075-12-1

**Published:** 2015-01-13

**Authors:** Jia-Yu Ke, Kara L Kliewer, Essam M Hamad, Rachel M Cole, Kimerly A Powell, Rebecca R Andridge, Shana R Straka, Lisa D Yee, Martha A Belury

**Affiliations:** Department of Human Sciences, Human Nutrition Program, College of Education and Human Ecology, The Ohio State University, Campbell Hall 302, 1787 Neil Avenue, Columbus, Ohio 43210 USA; Department of Dairy Science, Faculty of Agriculture, Cairo University, Giza, Egypt; Department of Biomedical Informatics, College of Medicine, The Ohio State University, Columbus, Ohio USA; Division of Biostatistics, College of Public Health, The Ohio State University, Columbus, Ohio USA; Department of Surgery, College of Medicine, The Ohio State University, Columbus, Ohio USA

**Keywords:** Naringenin, Menopause, Obesity, Insulin sensitivity, Adipose tissue inflammation, Fatty liver

## Abstract

**Objective:**

Adverse metabolic changes associated with loss of ovarian function increase the risk of developing metabolic syndrome and non-alcoholic fatty liver disease (NAFLD) in postmenopausal women. Naringenin improves metabolic disturbances in vitro and in vivo. In the present study, we tested the effects of naringenin on metabolic disturbances resulting from estrogen deficiency in ovariectomized mice.

**Materials/methods:**

Ovariectomized C57BL/6 J female mice were fed a control diet (10% calories from fat) for 11 weeks. Mice either continued on the control diet (n = 9) or were switched to the control diet supplemented with 3% naringenin (n = 10) for the next 11 weeks. Energy expenditure was measured by indirect calorimetry and activity was monitored by infrared beam breaks. Intra-abdominal and subcutaneous adiposity was evaluated by magnetic resonance imaging (MRI). Blood biochemical measures of metabolic response included glucose, insulin, adipokines, and lipids. Lipid content in liver and muscle and expression of relevant genes in adipose tissue, liver, and muscle were quantified.

**Results:**

Ovariectomized mice fed naringenin exhibited lower fasting glucose and insulin levels compared to controls, with over 50% reduction of intra-abdominal and subcutaneous adiposity. Plasma leptin and leptin mRNA in adipose depots were also decreased in mice fed a naringenin diet. Monocyte chemoattractant protein-1 (MCP1/Ccl2) and interleukin 6 (IL-6/Il6) mRNA expression levels were significantly lower in perigonadal adipose tissue of naringenin-supplemented mice. We also observed that mice fed a naringenin diet had less hepatic lipid accumulation with corresponding alterations of hepatic gene expression associated with de novo lipogenesis, fatty acid oxidation, and gluconeogenesis.

**Conclusion:**

Dietary naringenin attenuates many of the metabolic disturbances associated with ovariectomy in female mice.

**Electronic supplementary material:**

The online version of this article (doi:10.1186/1743-7075-12-1) contains supplementary material, which is available to authorized users.

## Introduction

During menopause, many women experience weight gain and accumulation of body fat in the waist region [1]. These changes of body composition increase the risk of developing metabolic syndrome [2], non-alcoholic fatty liver disease [3], and heart disease [4]. Although exogenous estrogen has been shown to be protective against many menopause-related metabolic abnormalities, long-term usage of hormone replacement therapy may increase the risk of breast cancer in addition to negative implications for cardiovascular diseases in postmenopausal women [5]. Lifestyle changes to reduce body weight, including healthy diet and regular exercise, are the initial strategies recommended for prevention and treatment of menopause-related metabolic disturbances.

Biologically active phytochemicals have attracted considerable attention for their potential health-promoting benefits [6, 7]. Naringenin is a flavonoid that is abundant in citrus fruits and tomatoes [8, 9]. Previously, we found that naringenin acts in a manner similar to metformin, a medicine used for treating type 2 diabetes, to reduce hepatic glucose production in hepatocytes [10]. In addition, naringenin improved some aspects of glucose and lipid homeostasis and mitigated adipose tissue inflammation *in vivo* [10–15]. However, the effects of naringenin on adipose depot mass and metabolic abnormalities associated with estrogen deficiency have not been studied.

Metabolic changes induced by estrogen depletion from ovariectomy share many similar characteristics with changes in menopausal women that are independent of energy intake, e.g., weight gain, increased adiposity, adipose tissue inflammation, and the development of fatty liver with inflammation [16, 17]. These similarities make ovariectomized mice a good model to study physiological changes after menopause. Therefore, in the present study we investigated the effect of 3% wt/wt naringenin supplementation in female ovariectomized (OVX) mice. The aim of our study is to determine whether naringenin ameliorates weight gain and attenuates accumulation of subcutaneous and abdominal adipose tissues, with resultant decreases in fasting glucose and ectopic lipid accumulation in muscle and liver of OVX mice. Our findings suggest that many of the effects of naringenin on dysregulated metabolism are related to effects of reduced adipose mass and ectopic lipid deposition in muscle and liver of OVX mice fed diet with naringenin.

## Materials and methods

### Animals and diets

Twenty-week old C57BL/6 J female mice were ovariectomized at 19 weeks old (Jackson Laboratory; Bar Harbor, ME, USA) and housed 4–5 per cage at 22 ± 0.5°C on a 12:12-h light–dark cycle. After 2 weeks of acclimation, mice (n = 19) were fed ad libitum a semi-purified diet for 11 weeks to enhance weight gain (D12450J, Research Diets Inc. New Brunswick, NJ, USA, formula is shown in Additional file 1: Table S1). Then mice were randomized by weight into either CON (n = 9) or NAR (n = 10) group (randomization was based on 19 mice as one mouse died prior to assignment to diet groups). For the next 11 weeks, the CON group continued on the control diet and the NAR group received the control diet supplemented with 3% wt/wt naringenin (Sigma, St. Louis, MO, USA), custom prepared by Research Diets Inc. (formula is shown in Additional file 1: Table S1). This dose of naringenin is based on a previously published study showing amelioration of metabolic disturbances in C57BL/6 J male mice fed a high-fat diet with 3% naringenin [12]. Body weight and food intake were measured daily. At week 22, mice were fasted for 5 h, anesthetized with isoflurane for blood collection via cardiac puncture, and then euthanized by cervical dislocation. Blood was collected into EDTA-coated blood collection tubes and plasma was obtained after centrifugation. Tissues, including subcutaneous adipose tissue (SCAT, thoracic and abdominal mammary fat pads), liver, perigonadal adipose tissue (PGAT), and quadriceps skeletal muscle, were excised, weighed, snap frozen in liquid nitrogen, and stored at -80°C until further analysis. All procedures were in accordance with institution guidelines and approved by the Institutional Animal Care and Use Committee at The Ohio State University.

### Fasting glucose analysis

At weeks 5 and 18, glucose was measured from tail vein blood samples after a 5 h period of fasting (OneTouch Ultra blood glucose meter, LifeScan Inc., Milpitas, CA, USA).

### Plasma analysis

Plasma insulin, leptin, and adiponectin levels were measured by ELISA (Millipore, Billerica, MA, USA) according to the manufacturer’s instructions. Plasma triglycerides were examined using a Cholestech LDX analyzer (Cholestech Corporation, Hayward, CA, USA). Plasma free fatty acids (NEFA C, Wako Chemicals, Richmond, VA, USA) and total cholesterol (Pointe Scientific Inc., Canton, MI, USA) were determined by enzymatic colorimetric assays.

### Indirect calorimetry

During week 17, six mice in each group were housed individually in metabolic chambers at 22°C, allowed free access to food and water, and acclimated 24 hours prior to metabolic assessments. Measurements were taken for a 24-h period, including a 12-h light cycle and a 12-h dark cycle. Oxygen consumption (VO_2_), carbon dioxide production (VCO_2_), and physical activity (by infra-red beam breaks) were measured every 20 minutes using a computer-controlled, open-circuit Oxymax/CLAMS System (Columbus Instruments, Columbus, OH, USA). Respiratory exchange ratio (RER) was calculated as the ratio of VCO_2_ to VO_2_. Heat, the standard measure of energy expenditure, was calculated with the formula [18],


### Magnetic Resonance Imaging (MRI)

During week 19, total, intra-abdominal, and subcutaneous adiposity were analyzed by MRI using a Bruker Biospin 94/30 magnet (Billerica, MA, USA) and a 70 mm diameter linear volume coil. T1-weighted coronal images of the whole mouse torso were collected using a respiratory-gated RARE sequence (TR/TE = 1570/7.5 ms, RARE factor = 4, FOV = 70×45 mm^2^, matrix size = 256×192, slice thickness = 1 mm, navg = 2). The details and definition of fat areas are shown in Additional file 1: Figure S1.

### Histology

Liver samples were collected at necropsy and fixed in 10% neutral buffered formalin overnight and transferred to 70% ethanol for storage. Tissues were then processed, embedded in paraffin, sectioned, and stained with hematoxylin and eosin (H&E).

### Total lipids, triacylglyceride and diacylglyceride analysis of liver and muscle

Total lipids were extracted from liver or muscle samples with 2:1 (v/v) chloroform: methanol and washed with 0.88% KCL [19]. The chloroform phase was transferred to a weighed test tube and dried under nitrogen gas at room temperature. The test tube containing the dried sample was weighed again to calculate total extracted lipid. Following the lipid extraction, triacylglycerol and diacylglycerol were obtained using solid-phase extraction [20] and solubilized in tert-butanol, methanol, Triton X-100 [21]. Analysis was performed using enzymatic colorimetric assay [22].

### RNA extraction and quantitative real-time polymerase chain reaction (qRT-PCR) analysis of gene expression

Total RNA was extracted from liver and muscle samples with QIAzol lysis reagent (Qiagen, Valencia, CA, USA) and from perigonadal adipose tissue and subcutaneous adipose tissue using RNeasy Lipid Tissue Mini Kit (Qiagen) following manufacturer’s instructions. Total RNA was then reversed transcribed to cDNA using High Capacity cDNA Reverse Transcription Kit (Applied Biosystems, Foster City, CA, USA). qRT-PCR analysis was performed with ABI Prism 7300 sequence detection system (Applied Biosystems) using TaqMan Gene Expression Assays (Applied Biosystems, Additional file 1: Table S2). Target gene expression was normalized to 18S rRNA for perigonadal adipose tissue or glyceraldehyde 3-phosphate dehydrogenase (GAPDH) for liver, subcutaneous adipose tissue, and muscle. Endogenous Controls (VIC probes) were amplified in the same reaction and expressed as 2-ΔΔCt compared to the CON group [23].

### Statistical analysis

All data are presented as mean ± standard error (SEM) with p < 0.05 considered significantly different. Statistical analysis was performed using SPSS version 20.0 software (SPSS, Inc., Chicago, IL, USA). Significance was determined using two-tailed unpaired Student’s *t* test. Caloric intake, heat, and adipose mass were also analyzed by ANCOVA with body weight as a covariate [24]. Pearson correlation coefficient was used for the correlation analyses.

## Results

### Effect of naringenin on caloric intake, body weight, and metabolic measurements

**Figure 1:**
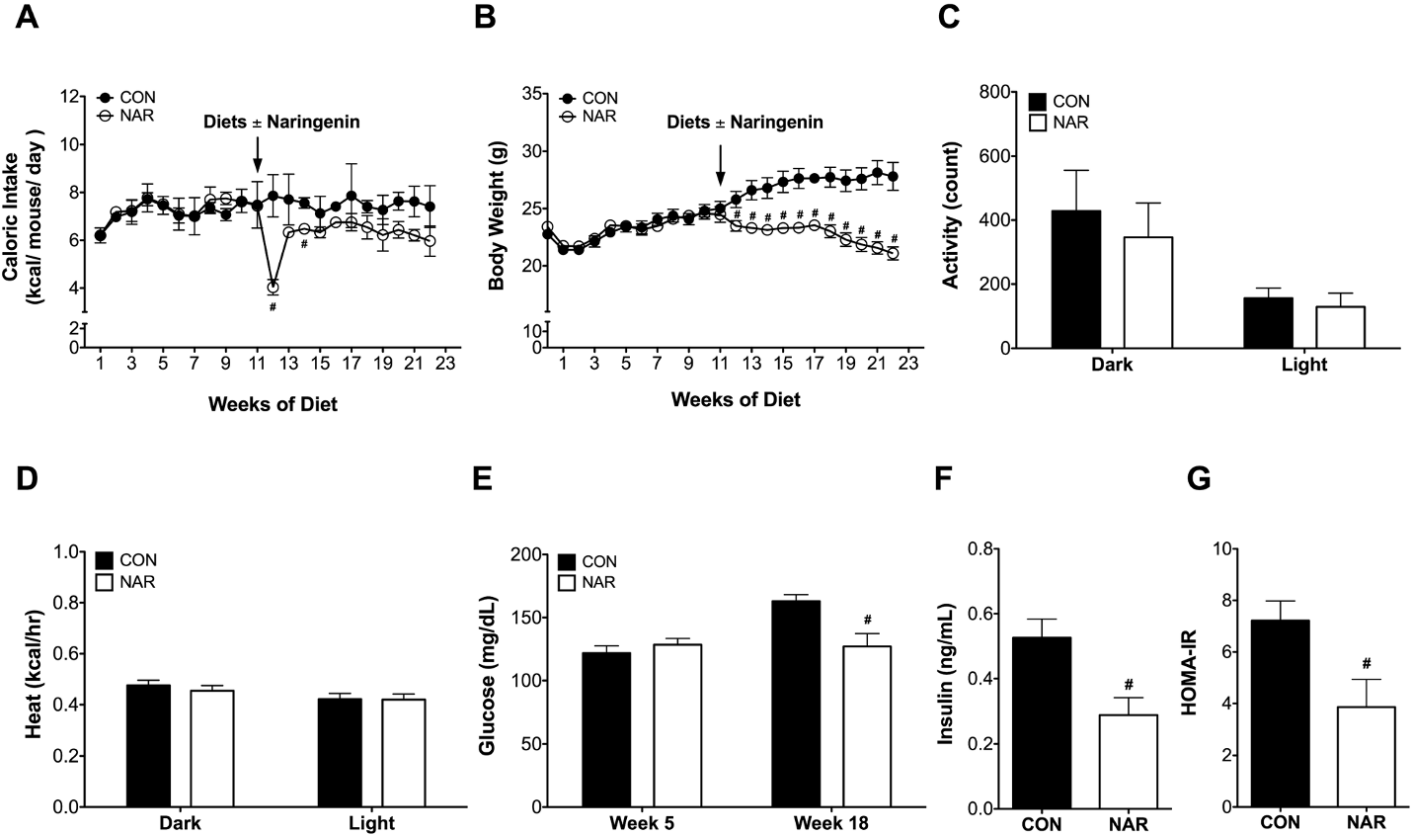
**Effects of dietary naringenin on caloric intake, body weight, and metabolic measurements.** OVX C57BL/6 J mice were fed the control diet for 11 weeks then randomized to continuation of the control diet (n = 9) or switched to 3% wt/wt naringenin supplementation of the control diet (n = 10) for the weeks 12–22. Average daily caloric intake **(A)** of two groups (n = 2/group) and weekly body weight (B) of the CON (n = 9) and NAR (n = 10) group were determined. At week 17, CLAMS chambers were used to measure locomotor activity (ambulation) in the horizontal plane by infrared beam breaks **(C)** and estimated energy expenditure (heat) after controlling for body weight **(D)** in the dark and light phase for 24-h following a 24-h acclimation (n = 6/group). Fasting glucose levels **(E)** were measured at week 5 and week 18, (CON, n = 9; NAR, n = 10). Fasting insulin levels **(F)** were determined after 22 weeks of experimental period (n = 6/group). HOMA-IR values **(G)** were derived from fasting plasma glucose and insulin (n = 4/group). Values are presented as mean ± SEM. Significance between groups was determined by Student’s t test, except metabolic data of heat was analyzed by ANCOVA with body weight as a covariate. ^#^P < 0.05 compared CON with NAR.

Caloric intake decreased significantly when mice were switched to a naringenin-containing diet at week 12 (Figure 1A) without complete recovery to baseline in the following weeks. However, excluding week 12, daily caloric intake from week 0–11 to week 13–22 did not differ significantly between the two treatment groups (p = 0.075). Body weights were significantly reduced in the NAR group from week 12 until the end of the study (Figure 1B). There were no differences in caloric intake between groups after adjusting for body weight (ANCOVA, p = 0.508). There were also no differences in ambulatory activity (Figure 1C) and energy expenditure after controlling for body weight differences between groups (Figure 1D).

Fasting glucose levels were significantly elevated in the CON mice from 121.8 ± 5.8 mg/dl at week 5 to 163.0 ± 5.2 mg/dl at week 18 (Figure 1E), while unchanged in NAR mice (128.6 ± 4.8 at week 5 and 127.1 ± 10.2 at week 18). Additionally, diets enriched with naringenin resulted in decreased fasting insulin levels and HOMA-IR values (Figure 1F & G), surrogate markers of insulin sensitivity. Using an insulin tolerance test, we observed that glucose levels were significantly lower in the NAR mice compared to the CON mice at 45 min (48.0 ± 3.4 mg/dl vs. 64.8 ± 8.1 mg/dl) and 60 min (51.4 ± 8.2 mg/dl vs. 77.0 ± 6.1 mg/dl) after injection of insulin (Additional file 1: Figure S2). However, no difference was observed in areas under the curve (AUC) between groups.

### Effect of naringenin on adiposity, plasma adipokines, and adipose tissue gene expression

**Figure 2:**
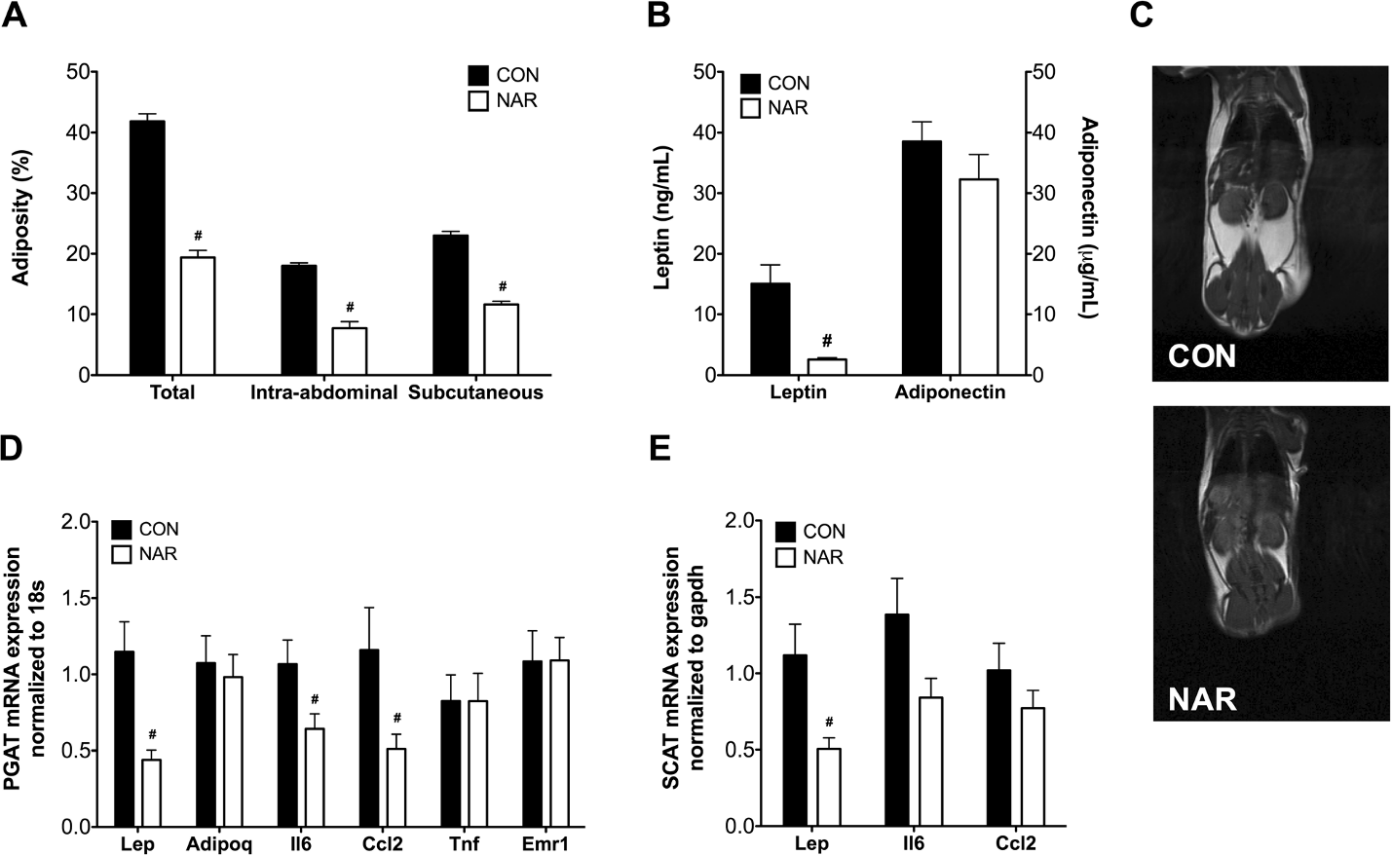
**Effects of dietary naringenin on adiposity, plasma adipokines, and adipose tissue gene expression.** At week 19, MRI analysis was performed in a subset of mice (n = 6/group) to measure percentage of total body fat and percentage of two adipose tissue depots, intra-abdominal and subcutaneous adipose tissues **(A)**. Fasting plasma leptin and adiponectin in the CON (n = 6) and NAR (n = 5-6) groups were measured at the end of study at 22 weeks **(B)**. Panel **(C)** shows representative coronal MRI views from each group. Effects of dietary naringenin on the mRNA levels of genes in perigonadal adipose tissue **(D)** and in subcutaneous adipose tissue **(E)** are shown (CON, n = 6-8; NAR, n = 10). Values are presented as mean ± SEM. Significance between groups was determined by Student’s t test. ^#^P < 0.05 compared CON with NAR.

MRI analysis was performed in a subset of mice from both groups (n = 6), which revealed a reduction in total, intra-abdominal and subcutaneous adiposity by 54, 59, and 50%, respectively, in mice fed naringenin versus control diets (Figure 2A & C). Additionally, naringenin was significantly associated with decreased perigonadal (PGAT) and subcutaneous (SCAT) adipose tissues (Table 1). Because body weights differed significantly between groups and body weight had a significant effect on perigonadal and subcutaneous adipose tissue mass (p < 0.001), we used ANCOVA to adjust for this difference when comparing adipose tissue mass. Mice fed naringenin had significantly decreased perigonadal adipose tissue mass (p = 0.006) after controlling for body weight in comparison to control mice. Fasting plasma leptin levels decreased by 80% in the NAR mice (Figure 2B) and strongly correlated with total, intra-abdominal, and subcutaneous adiposity determined by MRI (total, r = 0.85, p = 0.008; intra-abdominal, r = 0.86, p = 0.006; subcutaneous, r = 0.80, p = 0.016), as well as both perigonadal and subcutaneous adipose tissue mass (r = 0.95 and 0.97 respectively, both p < 0.001). Interestingly, plasma leptin levels correlated positively with insulin levels (r = 0.76, p = 0.004), as previously shown in women [25]. Dietary naringenin was not associated with changes in levels of adiponectin in plasma and mRNA expression (Adipoq) in perigonadal adipose tissue between groups (Figure 2B & D). Mice fed naringenin also had significantly lower leptin mRNA expression (Lep) in adipose depots, with a 60% reduction in perigonadal adipose tissue and a 55% reduction in subcutaneous adipose tissue (Figure 2D & E).

We measured mRNA levels of genes encoding for several markers of inflammation related to obesity, including the chemokine MCP1 (Ccl2), proinflammatory cytokine IL6 (Il6) and TNFα (Tnf), and macrophage-specific marker F4/80 (Emr1) in adipose depots (Figure 2D & E). The diet with naringenin significantly down-regulated mRNA levels of MCP1 (56% reduction) and IL6 (40% reduction) in perigonadal adipose tissue, but had no significant effect on MCP1 and IL6 levels in subcutaneous adipose tissue. TNFα and F4/80 mRNA was not affected by the naringenin supplementation in perigonadal adipose tissue.

**Table 1:** **Effect of dietary naringenin on tissue mass in OVX C57BL/6 J female mice**

	CON	NAR
**Tissue Weights (g)**		
Perigonadal Adipose Tissue	1.44 ± 0.15	0.38 ± 0.05^#^
Subcutaneous Adipose Tissue	1.21 ± 0.14	0.52 ± 0.03^#^
Liver	0.94 ± 0.05	0.80 ± 0.03^#^
Muscle (Quadriceps)	0.36 ± 0.01	0.32 ± 0.02
**Tissue Weight Percentages (%)**		
Perigonadal Adipose Tissue	5.23 ± 0.35	1.81 ± 0.19^#^
Subcutaneous Adipose Tissue	4.34 ± 0.34	2.55 ± 0.15^#^
Liver	3.45 ± 0.09	3.89 ± 0.12^#^
Muscle (Quadriceps)	1.33 ± 0.07	1.55 ± 0.07^#^

Significance between groups was determined by Student’s t-test (n = 9–10). Data represent the mean ± SEM.

^#^P < 0.05 compared NAR with CON.

### Effect of naringenin on plasma, hepatic, and muscle lipid profile

**Figure 3:**
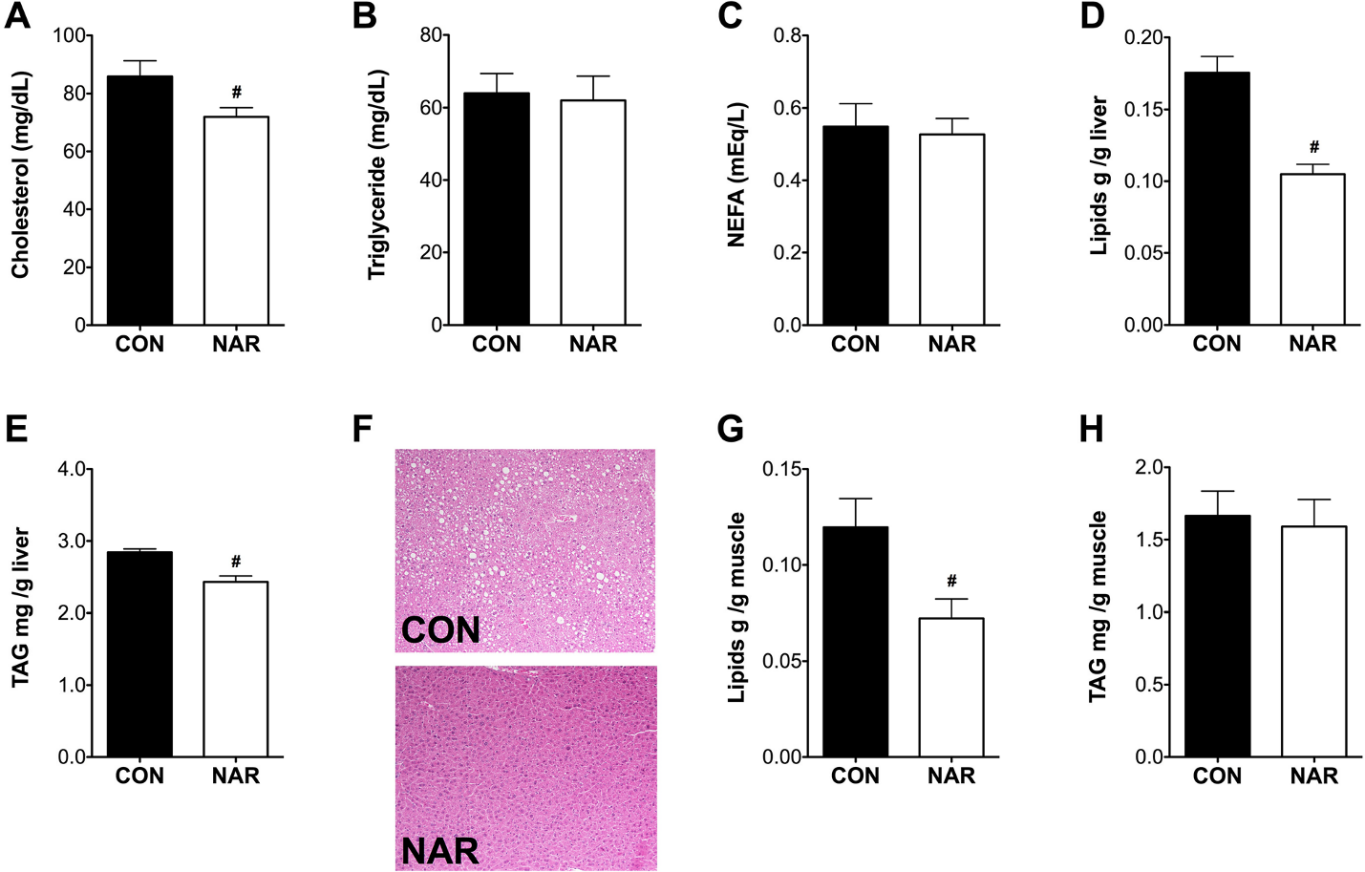
**Effects of dietary naringenin on plasma lipids and lipid accumulation in liver and muscle.** Plasma cholesterol (**A**; CON, n = 6; NAR, n = 10), triglyceride (**B**; n = 4/group) and NEFA (**C**; n = 6/group) after 5-h fasting obtained prior to necropsy were measured by enzymatic colorimetric method. Total lipids (**D**; CON, n = 9; NAR n = 9) and triglyceride (**E**; CON, n = 8; NAR n = 9) per gram of liver section were determined. Panel **(F)** shows representative H&E staining of livers (10x) from each group. Total lipids **(G)** and triglyceride **(H)** per gram of muscle section were also determined (CON, n = 9; NAR n = 9). Values are presented as mean ± SEM. Significance between groups was determined by Student’s t test. ^#^P < 0.05 compared CON with NAR.

Dietary naringenin reduced total cholesterol levels (Figure 3A), but did not change the levels of triglyceride and non-esterified fatty acids in plasma (Figure 3B & C). As ectopic lipid accumulation in liver has been observed in OVX animals [26], we tested hepatic lipid contents in both groups. Dietary naringenin decreased the extracted total lipids and triacylglyceride levels in the liver (Figure 3D & E) but did not affect diacylglyceride levels (data not shown). Small lipid droplets were scattered throughout H&E stained liver sections from the CON group, while no lipid droplets were detected in the NAR group (Figure 3F). OVX has been shown to increase muscle lipid contents [27]. Therefore, we examined muscle lipid levels and found naringenin reduced the extracted total lipids levels (Figure 3G) but had no effect on either triacylglyceride (Figure 3H) or diacylglyceride levels in skeletal muscle (data not shown).

### Effect of naringenin on hepatic and muscle mRNA expression

**Figure 4:**
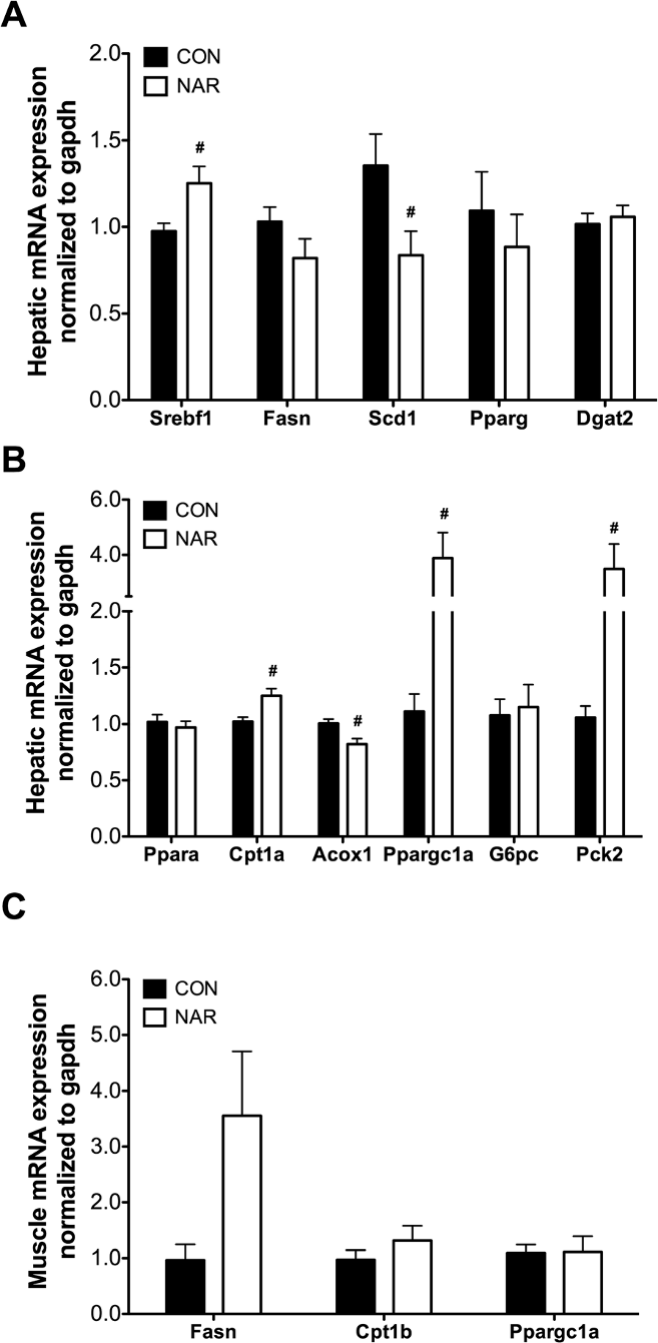
**Effects of dietary naringenin on gene expression in liver and muscle.** mRNA expression of hepatic genes related to de novo lipogenesis and hepatic steatosis **(A)**, mRNA expression of hepatic genes related to beta-oxidation and gluconeogenesis **(B)**, and mRNA expression of genes related to de novo lipogenesis and beta-oxidation in muscle **(C)** were quantified using qRT- PCR (n = 6-10/group). Values are presented as mean ± SEM. Significance between groups was determined by Student’s t test. ^#^P < 0.05 compared CON with NAR.

Srebf1, Fasn, and Scd1 encode proteins involved in *de novo* lipogenesis. Increased hepatic expression of Srebf1 was observed in the NAR mice (Figure 4A). However, naringenin down-regulated Scd1 mRNA by 38% but had no effect on another lipogenic enzyme, Fasn. No differences in mRNA levels of genes related to steatotic liver [28, 29] were detected, e.g., Pparg and Dgat2 (Figure 4A). Expression of genes involved in fatty acid oxidation, Cpt1α (mitochondrial) was significantly higher in the mice fed a naringenin diet, but Acox1 (peroxisomal) was decreased (Figure 4B). PGC1α (Ppargc1a) is a transcriptional coactivator regulating genes involved in fatty acid oxidation and gluconeogenesis. Dietary naringenin induced PGC1α mRNA (4-fold) as well as PEPCK (Pck2) mRNA (3.5-fold), a rate-limiting enzyme in hepatic gluconeogenesis (Figure 4B), but not G6Pase (G6pc) mRNA, another enzyme involved in gluconeogenesis. Having observed reduced lipid content in skeletal muscle, we measured the mRNA levels of genes involved in fatty acid metabolism (Figure 4C). However, naringenin had no effect on the expression of Cpt1β and PGC1α in muscle. Interestingly, Fasn mRNA expression was higher in muscle tissue of NAR relative to CON mice but did not reach significance (p = 0.07).

## Discussion

Estrogen deficiency leads to metabolic changes and increased risk of metabolic syndrome [1–3, 30]. To our knowledge, this is the first paper to examine the effects of naringenin on energy metabolism and adiposity in OVX female mice, a preclinical model of menopause. We report here that OVX mice exhibited metabolic disturbances expected with estrogen deficiency including elevated fasting glucose and obesity [3, 26, 31]. The addition of naringenin to the diet 1) decreased body weight, fasting glucose and insulin, 2) reduced body fat and perigonadal adipose tissue inflammation, 3) decreased plasma cholesterol and ectopic lipid accumulation in liver and muscle, and 4) affected expression of hepatic genes involved in de novo lipogenesis and fatty acid oxidation. Therefore, our study results suggest that a 3% wt/wt dietary naringenin supplementation ameliorates many metabolic derangements associated with estrogen deficiency.

We observed that naringenin supplementation caused an initial reduction of caloric intake, followed by a decrease in caloric intake and body weight over the subsequent ten weeks of feeding. Reduced caloric intake after naringenin supplementation in the present study was unexpected because previous studies had reported no differences in food intake in male rodent models supplemented with naringenin [11–13, 32–34]. No changes in food intake were observed even in the same strain, wild-type C57BL/6 J males [12, 35]. Discrepancies between our data from studies in C57BL/6 J males may be attributed to the fat content in the diet, age of the mice, and different effects of naringenin on appetite between male and female OVX mice.

Estrogen interacts with neuropeptides, decreases food intake, and reduces weight gain [36]. Hyperphagia has been reported in some models of menopause [37] but not all [17, 36–38]. Instead of altering food intake, several studies demonstrated that OVX mice have lower energy expenditure and activity levels, especially in the dark phase of the light/dark cycle [17, 37–39]. These findings are consistent with human data demonstrating decreased free-living and 24 h energy expenditure, and reduced physical activity during the menopausal transition [40]. The totality of evidence suggests that reduced energy expenditure and increased energy efficiency, rather than overeating, results in the menopause-associated metabolic disturbances. We did not observe differences in spontaneous physical activity and in energy expenditure between groups after controlling for body weight. It is unclear whether the effects of naringenin on body weight and other OVX-related metabolic disturbances are attributed to reduced caloric intake and/or other mechanisms. In future studies, a pair-fed group will be included to control for possible differences in energy intake as a potential confounding factor.

Dietary naringenin attenuated hyperglycemia and hyperinsulinemia induced by a high-fat or a fructose-enriched diet [12, 33]. We observed development of hyperglycemia in OVX mice from week 5 to week 18, consistent with Roger et al. who showed elevated fasting glucose in mice after 12 weeks of ovariectomy compared to sham-operated mice [16], while naringenin supplementation prevented the development of hyperglycemia and lowered fasting insulin concentration and HOMA-IR value. Potter *et al.* suggest that a significant difference in insulin resistance (determined by insulin tolerance test) may not develop until as late as 26 weeks post-ovariectomy [17], which may explain why insulin reduced blood glucose levels more effectively in mice supplemented with naringenin but the glucose AUC did not reach significance in the present study.

Estrogen deficiency increases the susceptibility to weight gain and central obesity in both humans and mice [11, 16, 41]. Hong *et al.* demonstrated that adiposity in OVX female mice, examined by DEXA, were comparable with those of male mice [42]. Additionally, despite consuming a low-fat diet, the OVX mice accumulated about 40% body fat and about a 25% reduction in percent body fat after undergoing a 30% calorie restriction [42]. Similarly, we found that OVX mice accumulated about 40% of body fat as assessed by MRI. Although naringenin reduced caloric intake by ~14%, total adiposity decreased by approximately 50%. Additionally, naringenin significantly reduced perigonadal adipose tissue mass, even after controlling for body weight. Dietary naringenin has been linked to decreased adiposity in several male mouse studies independent of caloric intake [12, 34, 35]. Such data further suggest that the effect of naringenin on adiposity is not simply attributable to lower caloric intake.

Increased visceral fat and pro-inflammatory activity have been observed in postmenopausal women [43, 44]. Previous studies indicate adipose tissues inflammation occurred early in OVX mice (e.g., 12 weeks after ovariectomy) and progressively worsens as indicated by increased infiltration and activation of immune cells and decreased insulin sensitivity [16, 17]. Genetic deletion of MCP1 reduces body fat, increases glucose tolerance, and ameliorates adipose inflammation in visceral fat pad in OVX mice, but has no effect on sham-operated mice. These findings suggest that MCP1 may be a mediator of OVX-induced metabolic disturbances attributed to adipose tissue inflammation [45]. Yoshida et al. demonstrated that naringenin inhibited high-fat-diet induced toll-like receptor 2 mRNA expression and suppressed mRNA levels of proinflammatory mediators, TNFα and MCP1, in epididymal/perigonadal adipose tissue [35]. We found that naringenin down-regulated mRNA levels of MCP1 but not TNFα in perigonadal fat. Future studies will investigate the mechanisms of naringenin action on adipose tissue inflammation of OVX mice.

Interestingly, we found that naringenin significantly reduced MCP1 and IL6 mRNA in perigonadal adipose tissue, but had no significant effect on these markers of inflammation in subcutaneous adipose tissue. Rogers *et al.* [16] suggested that adipose tissue inflammation associated with OVX is more severe in perigonadal adipose tissue than in subcutaneous adipose tissue, which may explain why we found a more significant effect of naringenin on perigonadal adipose tissue inflammation. Additionally, by LC/MS-MS analyses, naringenin accumulated 1.7 fold more in perigonadal adipose than in subcutaneous adipose tissue after 11 weeks of 3% naringenin supplementation (3.03 ± 2.00 μmole/kg vs. 5.11 ± 1.26 μmole/kg, data unpublished), suggesting that naringenin may have less influence on subcutaneous adipose tissue.

Estrogen deficiency has been connected to hepatic fat accumulation in women and rodents [3, 26]. In the present study, we demonstrated that naringenin supplementation led to decreased hepatic lipid accumulation and changed mRNA levels of some genes involved in de novo lipogenesis and fatty acid oxidation. Several studies have shown that naringenin induces PPARα activity and downstream enzymes involved in fatty acid oxidation, such as CPT1α, UCP2, and ACOX1 [11, 13]. Interestingly, we observed increased Cpt1α mRNA levels but decreased Acox1 levels, implicating discordance in mitochondrial and peroxisomal β-oxidation in response to OVX and naringenin treatment. Goldwasser *et al.* [14] also showed that naringenin induced a fasted-like state in hepatocytes, inhibiting fatty acid and cholesterol synthesis and increasing fatty acid oxidation. Consistent with these *in vitro* results, we also observed increased Srebf1 and PGC1α mRNA in the liver of mice supplemented with naringenin. However, we did not observe increased expression microsomal triglyceride transfer protein (Mttp) expression and were unable to detect low-density lipoprotein (LDL) receptor mRNA expression (data not shown), which are downstream genes of Srebf1 and inducible by naringenin *in vitro* [46]. Additionally, we found increased hepatic expression of genes controlling gluconeogenesis in the mice fed naringenin, a normal physiological reaction in response to fasting. Lack of induction of these two genes in the CON mice may be related to their high levels of fasting glucose.

We demonstrated that naringenin decreased plasma cholesterol but did not change the levels of plasma triacylglyceride and non-esterified fatty acids in OVX mice. However, the effects of OVX on blood triacylglyceride and non-esterified fatty acids are mixed. Several studies have shown no difference in blood triacylglyceride and non-esterified fatty acids between sham-operated and OVX rodents [16, 17, 39, 47–49], while others have observed increased levels in OVX rodents [27, 50]. It is possible that OVX had no effect on triacylglyceride and non-esterified fatty acids in the current study. Therefore we did not observe changes in plasma triacylglyceride and non-esterified fatty acids in naringenin-supplemented mice.

Estrogen deficiency-associated metabolic disturbances have been widely demonstrated in OVX mice [16, 17, 37, 39, 40, 42, 51]. There were several limitations of the present study. Due to the atrophy of the uterus observed in our OVX mice, we could not measure the weight of uterus to determine whether there is an estrogenic or anti-estrogenic effect of naringenin. Additionally, we did not have sham-operated mice as a control to evaluate the degrees of metabolic disturbances in our OVX mice and thus the extent of naringenin effects. However, the elevated fasting glucose, adiposity, and hepatic lipid accumulation observed in OVX mice in the present study were markedly reduced with naringenin supplementation. In addition, as mentioned earlier, we did not design this study to include a pair-fed group since previous studies evaluating metabolic effects of diets with naringenin in male rodents did not observe differences in food intake.

Naringenin readily accumulates in plasma after ingestion of orange juice, grapefruit juice [52], and tomato paste or sauce [53], suggesting that it is bioavailable in individuals who consume naringenin food sources regularly. In the present study, we found that mice developed higher fasting glucose, adiposity, and hepatic steatosis after loss of ovarian function, similar to what has been observed in postmenopausal women [3, 31]. Naringenin supplementation attenuated these estrogen-deficiency-associated metabolic disturbances in OVX female mice, suggesting the potential influence of dietary naringenin on metabolic syndrome in postmenopausal women. Further work in pre-clinical and human intervention studies will help to determine if naringenin is able to protect against menopausal-associated metabolic syndrome in humans.

## Electronic supplementary material

Additional file 1: Table S1: Composition of experimental diets. **Table S2:** Real-time PCR primers and probes of Taqman gene expression assay. **Figure S1:** Illustration of different fat depots analyzed in the present paper. Total, intra-abdominal, and subcutaneous adiposity were analyzed by MRI using a Bruker Biospin 94/30 magnet (Billerica, MA, USA) and a 70 mm diameter linear volume coil. T1-weighted coronal images of the whole mouse torso were collected using a respiratory-gated RARE sequence (TR/TE=1570/7.5ms, RARE factor=4, FOV=70x45 mm^2^, matrix size=256x192, slice thickness=1 mm, navg=2). Mice were anesthetized with 2-2.5% isoflurane mixed with 1 liter per minute carbogen (95%O_2_+5%CO_2_) and maintained with 1-1.5% isoflurane. Physiologic parameters such as the electrocardiography, respiration and the temperature of the animals were monitored using a small animal monitoring system (Model 1025, Small Animals Instruments, Inc. Stony Brook, NY, USA). Otsu segmentation [1] was used to segment the mouse body from background. A connected components algorithm [2] was used to label the background objects in the image and 'fill’ any holes in the segmented body image. The abdominal cavity was manually outlined in the images and a global threshold of 120 grey level intensity was chosen to segment fat from surrounding tissue. The whole body and abdominal masks were then used to calculate the percentage of segmented voxels in the whole body and abdomen, respectively. Subcutaneous fat was calculated by subtracting intra-abdominal fat from total fat. **Figure S2:** Insulin tolerance test was performed at week 21 to determine insulin sensitivity (CON, n=4; NAR n=5). After a 5h-fast, insulin was administered intraperitoneally at a dose of 0.4 U/kg body weight (Humulin R, Eli Lilly and Co., Indianapolis, IN, USA). Glucose was measured from tail vein blood using a OneTouch Ultra blood glucose meter immediately prior to insulin injection (time 0) and 15, 30, 45, 60, 90, and 120 minutes following the injection. The values were normalized to baseline (time 0) values and the percent change from baseline was plotted over time. The response curve was used to calculate area under the curve (AUC) [3]. Graphical representation of the calculated area under curve (AUC) between time point 0 to 120 min was shown on the top right. (DOCX 1 MB)

## Abbreviations

CON: Control diet group
HOMA-IR: Homeostasis model assessment of insulin resistance
NAR: Naringenin diet group
OVX: Ovariectomized
PGAT: Perigonadal adipose tissue
SCAT: Subcutaneous adipose tissue.
