# Supplementary material for: The flavonoid, naringenin, decreases adipose tissue mass and attenuates ovariectomy-associated metabolic disturbances in mice
Source: Nutr Metab (Lond). 2015 Jan 13;12:1. doi: 10.1186/1743-7075-12-1 (PMC4350282; doi:10.1186/1743-7075-12-1)
Supplement: Supplementary file 1 — Additional file 1: Table S1: Composition of experimental diets. Table S2: Real-time PCR primers and probes of Taqman gene expression assay. Figure S1: Illustration of different fat depots analyzed in the present paper. Total, intra-abdominal, and subcutaneous adiposity were analyzed by MRI using a Bruker Biospin 94/30 magnet (Billerica, MA, USA) and a 70 mm diameter linear volume coil. T1-weighted coronal images of the whole mouse torso were collected using a respiratory-gated RARE sequence (TR/TE=1570/7.5ms, RARE factor=4, FOV=70x45 mm2, matrix size=256x192, slice thickness=1 mm, navg=2). Mice were anesthetized with 2-2.5% isoflurane mixed with 1 liter per minute carbogen (95%O2+5%CO2) and maintained with 1-1.5% isoflurane. Physiologic parameters such as the electrocardiography, respiration and the temperature of the animals were monitored using a small animal monitoring system (Model 1025, Small Animals Instruments, Inc. Stony Brook, NY, USA). Otsu segmentation [1] was used to segment the mouse body from background. A connected components algorithm [2] was used to label the background objects in the image and 'fill’ any holes in the segmented body image. The abdominal cavity was manually outlined in the images and a global threshold of 120 grey level intensity was chosen to segment fat from surrounding tissue. The whole body and abdominal masks were then used to calculate the percentage of segmented voxels in the whole body and abdomen, respectively. Subcutaneous fat was calculated by subtracting intra-abdominal fat from total fat. Figure S2: Insulin tolerance test was performed at week 21 to determine insulin sensitivity (CON, n=4; NAR n=5). After a 5h-fast, insulin was administered intraperitoneally at a dose of 0.4 U/kg body weight (Humulin R, Eli Lilly and Co., Indianapolis, IN, USA). Glucose was measured from tail vein blood using a OneTouch Ultra blood glucose meter immediately prior to insulin injection (time 0) and 15, 30, 45, 60, 90, and 120 minutes follo [file 12986_2014_628_MOESM1_ESM.docx]

**Supplementary Table 1**. Composition of experimental diets

|  | **CON**  **(D12450J)** | | **NAR** | |
| --- | --- | --- | --- | --- |
| **Nutrient** | gm% | kcal% | gm% | kcal% |
| Protein | 19.2 | 20 | 18.7 | 20 |
| Carbohydrate | 67.3 | 70 | 65.3 | 70 |
| Fat | 4.3 | 10 | 4.1 | 10 |
| **kcal/g** | 3.85 |  | 3.73 |  |
|  |  |  |  |  |
| **Ingredient** | gm | kcal | gm | kcal |
| Casein | 200 | 800 | 200 | 800 |
| L-Cystine | 3 | 12 | 3 | 12 |
| Corn Starch | 506.2 | 2024.8 | 506.2 | 2024.8 |
| Maltodextrin 10 | 125 | 500 | 125 | 500 |
| Sucrose | 68.8 | 275.2 | 68.8 | 275.2 |
| Cellulose, BW200 | 50 | 0 | 50 | 0 |
| Soybean Oil | 25 | 225 | 25 | 225 |
| Lard | 20 | 180 | 20 | 180 |
| Mineral Mix S10026 | 10 | 0 | 10 | 0 |
| DiCalcium Phosphate | 13 | 0 | 13 | 0 |
| Calcium Carbonate | 5.5 | 0 | 5.5 | 0 |
| Potassium Citrate, H2O | 16.5 | 0 | 16.5 | 0 |
| Vitamin Mix V10001 | 10 | 40 | 10 | 40 |
| Choline Bitartrate | 2 | 0 | 2 | 0 |
| Naringenin | 0 | 0 | 32.7 | 0 |
|  |  |  |  |  |
| **Naringenin (%)** | 0 |  | 3.01 |  |

**Supplementary Table 2**. Real-time PCR primers and probes of Taqman gene expression assay

| **Gene** | **No.** |
| --- | --- |
| Acox1 | Mm00443579_m1 |
| Adipoq (Adiponectin) | Mm00456425_m1 |
| Ccl2 (MCP1) | Mm00441242_m1 |
| Cpt1a | Mm00550438_m1 |
| Cpt1b | Mm00487200_m1 |
| Dgat2 | Mm01273905_m1 |
| Emr1 (F4/80) | Mm00802529_m1 |
| Fas | Mm00662319_m1 |
| G6pc | Mm00839363_m1 |
| Il6 | Mm00446190_m1 |
| Lep (Leptin) | Mm00434759_m1 |
| Pck2 (PEPCK) | Mm00440636_m1 |
| Ppara | Mm00440939_m1 |
| Ppargc1a (PGC1a) | Mm00447183_m1 |
| Pparg | Mm00440945_m1 |
| Scd1 | Mm00772290_m1 |
| Srebf1 (SREBP1) | Mm00550338_m1 |
| Tnf (TNFa) | Mm00443258_m1 |

**Supplementary Figure 1.** Illustration of different fat depots analyzed in the present paper. Total, intra-abdominal, and subcutaneous adiposity were analyzed by MRI using a Bruker Biospin 94/30 magnet (Billerica, MA, USA) and a 70 mm diameter linear volume coil. T1-weighted coronal images of the whole mouse torso were collected using a respiratory-gated RARE sequence (TR/TE=1570/7.5ms, RARE factor=4, FOV=70x45 mm^2^, matrix size=256x192, slice thickness=1 mm, navg=2). Mice were anesthetized with 2-2.5% isoflurane mixed with 1 liter per minute carbogen (95%O_2_+5%CO_2_) and maintained with 1-1.5% isoflurane. Physiologic parameters such as the electrocardiography, respiration and the temperature of the animals were monitored using a small animal monitoring system (Model 1025, Small Animals Instruments, Inc. Stony Brook, NY, USA). Otsu segmentation [1] was used to segment the mouse body from background. A connected components algorithm [2] was used to label the background objects in the image and ‘fill’ any holes in the segmented body image. The abdominal cavity was manually outlined in the images and a global threshold of 120 grey level intensity was chosen to segment fat from surrounding tissue. The whole body and abdominal masks were then used to calculate the percentage of segmented voxels in the whole body and abdomen, respectively. Subcutaneous fat was calculated by subtracting intra-abdominal fat from total fat.

**
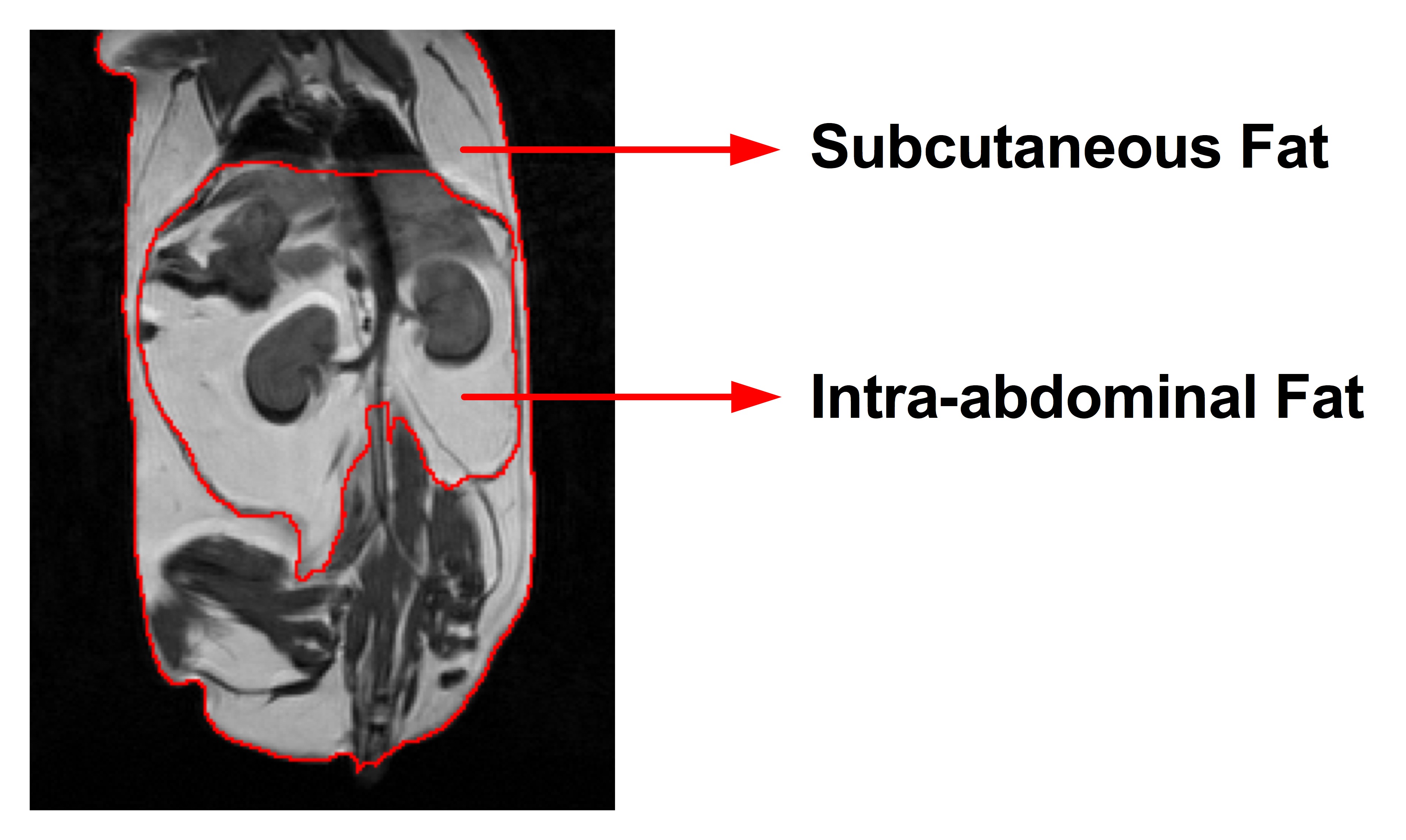
**

**Supplementary Figure 2.** Insulin tolerance test was performed at week 21 to determine insulin sensitivity (CON, n=4; NAR n=5). After a 5h-fast, insulin was administered intraperitoneally at a dose of 0.4 U/kg body weight (Humulin R, Eli Lilly and Co., Indianapolis, IN, USA). Glucose was measured from tail vein blood using a OneTouch Ultra blood glucose meter immediately prior to insulin injection (time 0) and 15, 30, 45, 60, 90, and 120 minutes following the injection. The values were normalized to baseline (time 0) values and the percent change from baseline was plotted over time. The response curve was used to calculate area under the curve (AUC) [3]. Graphical representation of the calculated area under curve (AUC) between time point 0 to 120 min was shown on the top right.

**
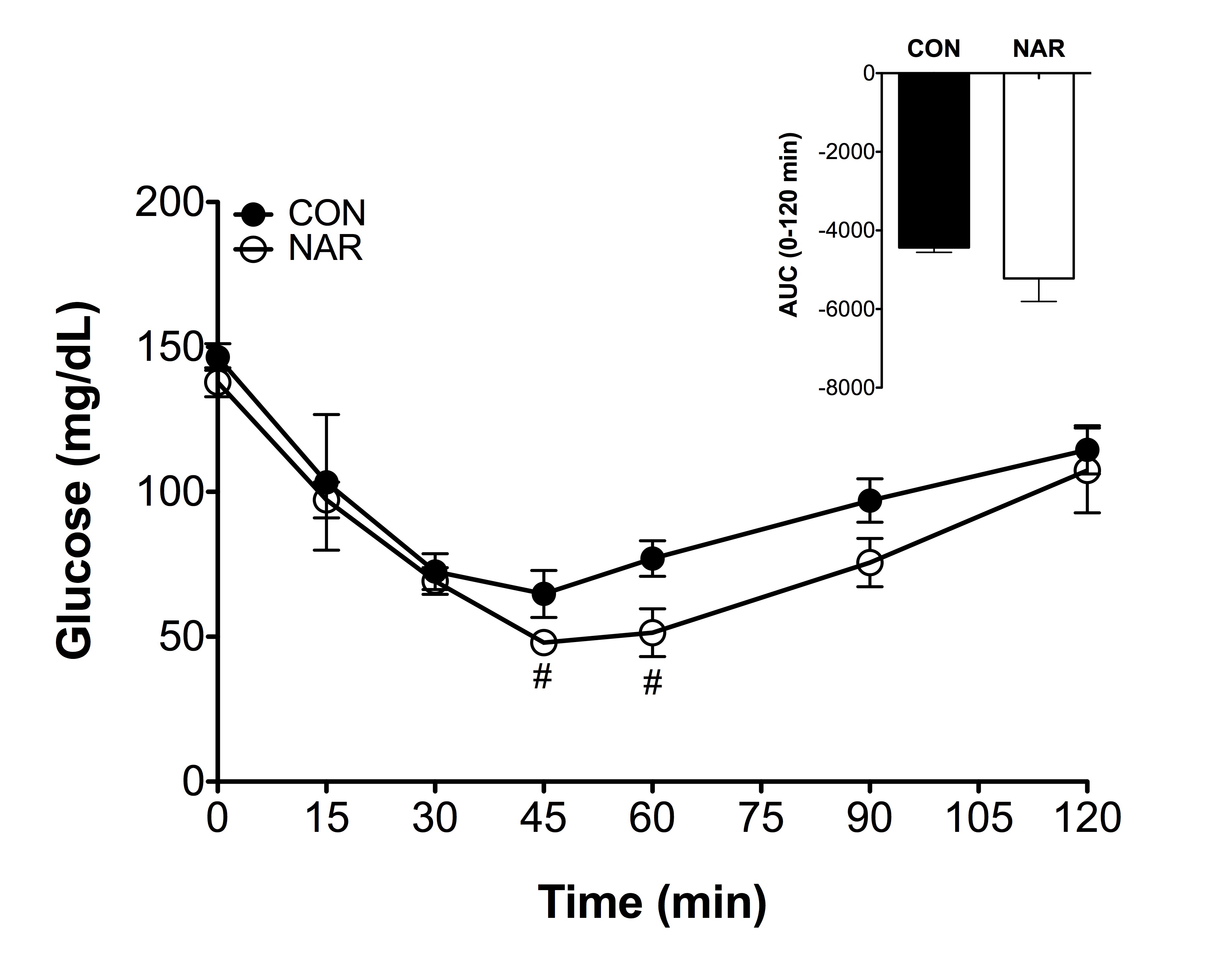
**

**References**

[1] Nakashima T, Arakawa M, Kojiro M*, et al.* Pathological and morphological studies of hepatocellular carcinoma, particularly on metastases and intracanal tumor growth. Kurume Med J. 1979; **26**: 21-33

[2] Gonzalez R, Woods R. Digital Image Processing. 3rd edn: Prentice Hall 2007.

[3] Wolever TM, Jenkins DJ, Jenkins AL, Josse RG. The glycemic index: methodology and clinical implications. Am J Clin Nutr. 1991; **54**: 846-854
